# Supplementary material for: Undenatured type II collagen protects against collagen-induced arthritis by restoring gut-joint homeostasis and immunity
Source: Commun Biol. 2024 Jul 3;7:804. doi: 10.1038/s42003-024-06476-z (PMC11222443; doi:10.1038/s42003-024-06476-z)
Supplement: Supplementary file 2 — Supplementary Information [file 42003_2024_6476_MOESM2_ESM.pdf]

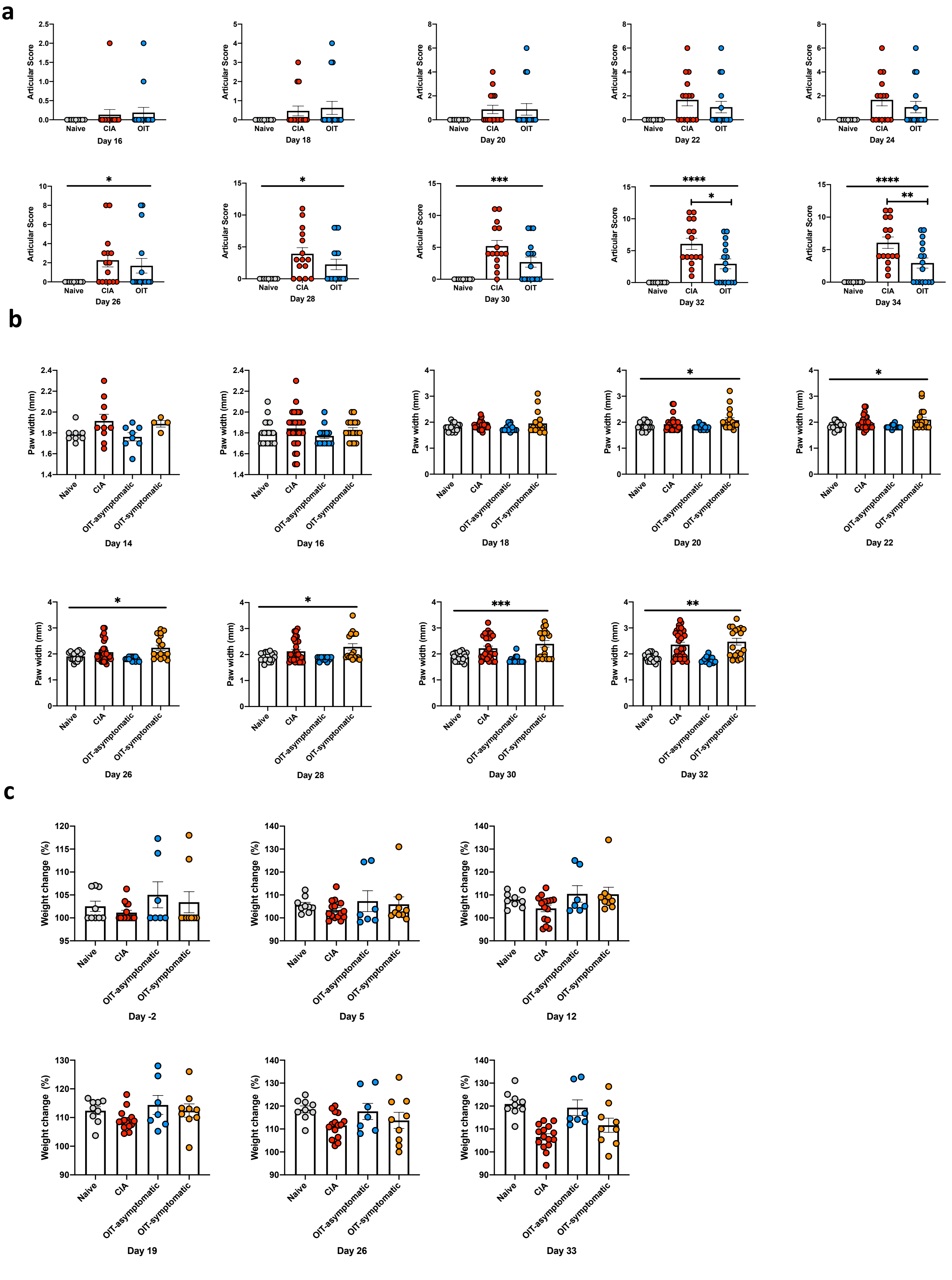

Supplementary figure 1. Disease scores and clinical data in response to oral immunotherapy (OIT) in CIA mice for individual mice. a) Disease scores, b) paw width and c) weight change, as in figure 1 a-c. Results show the numbers for each individual mouse represented in figure 1 as means. Each graph shows separated days after the initiation of the CIA model. Statistical significance was determined by one-way ANOVA; *p < 0.05, ***p < 0.01,***p < 0.001. Each dots represents one individual mouse.

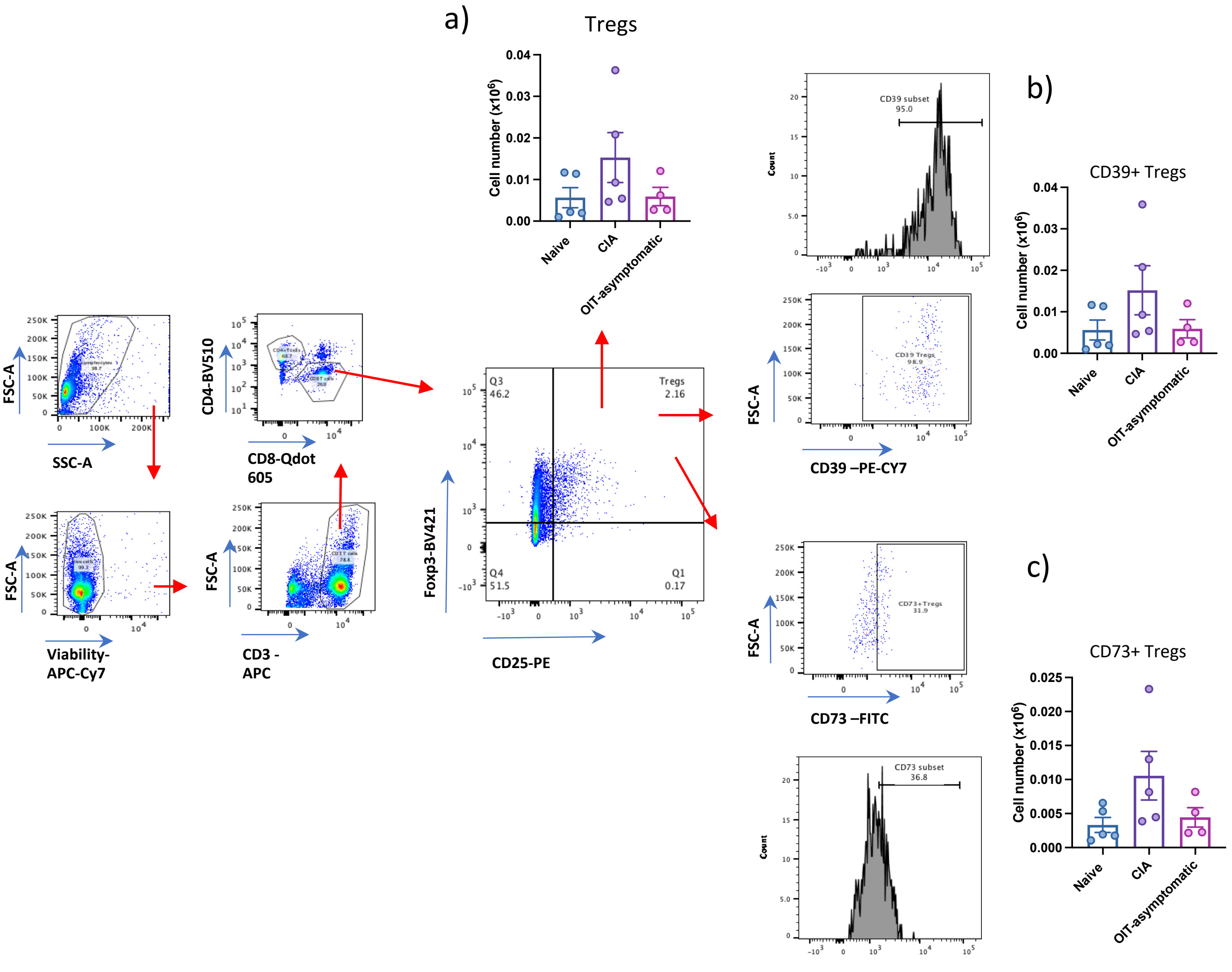

Supplementary figure 2. Analysis of regulatory T cell (Treg) populations in response to oral immunotherapy (OIT) in the joint. Gating strategy for flow cytometric analysis of total CD3+CD25+FoxP3+ Tregs (a) and specific CD39+ (b) and CD73+ (c) subsets. Column graphs show the total number of cells for naïve, CIA and asymptomatic OIT mice. For all panels, each dot in column bars represents individual mice from 2 independent experiments. Error bars show mean ± SEM.

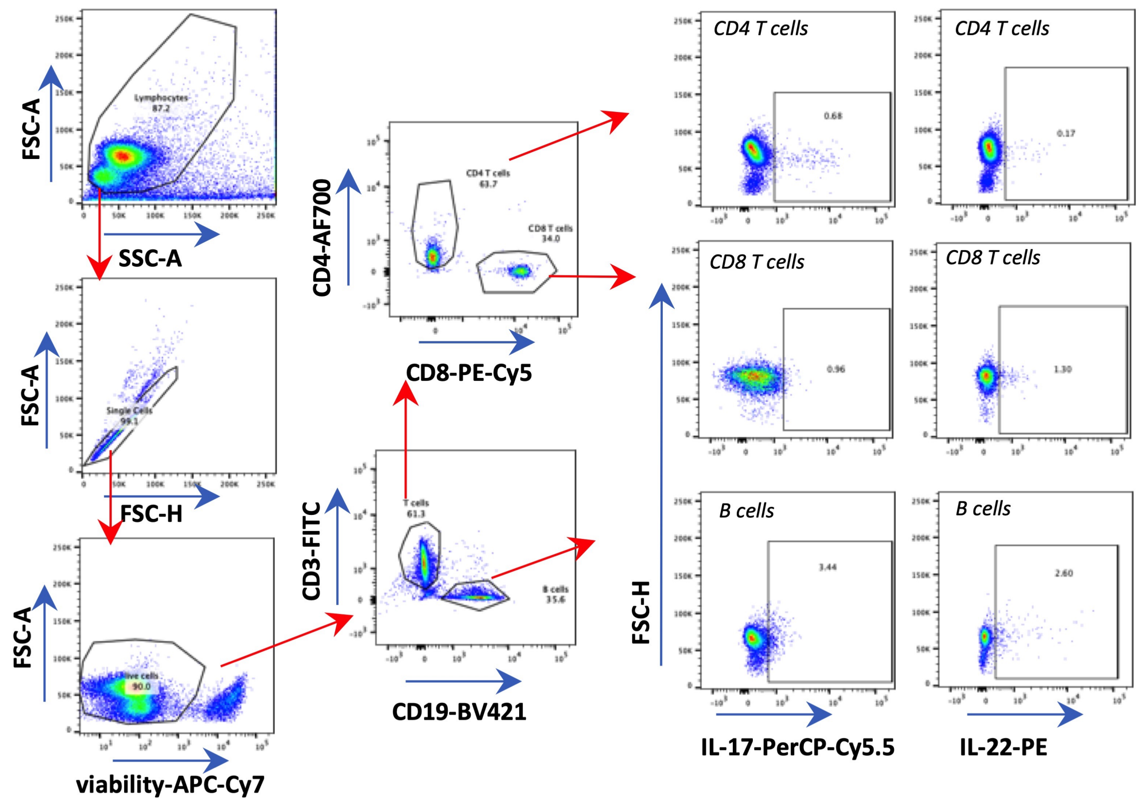

Supplementary figure 3. Analysis of IL-17 and IL-22 expression in CD4 T cells, CD8 T cells and B cells in DLNs. Gating strategy for flow cytometric analysis of IL-17+ and IL-22+ cells in CD3+CD4+ T cells, CD3+CD8+ T cells and CD3-CD19+ B cells in DLNs from a representative mouse.

a

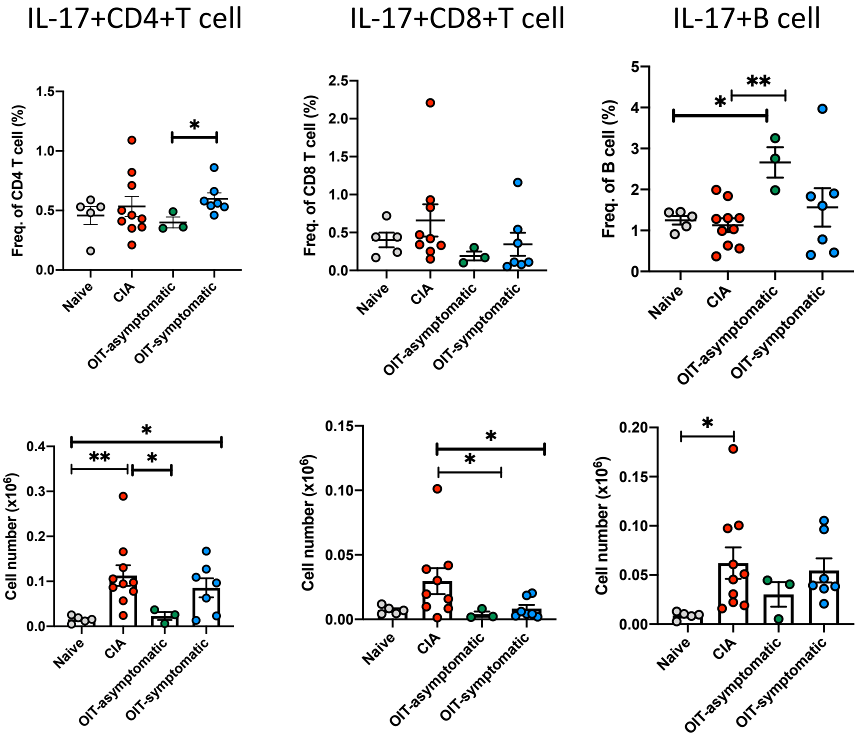

b

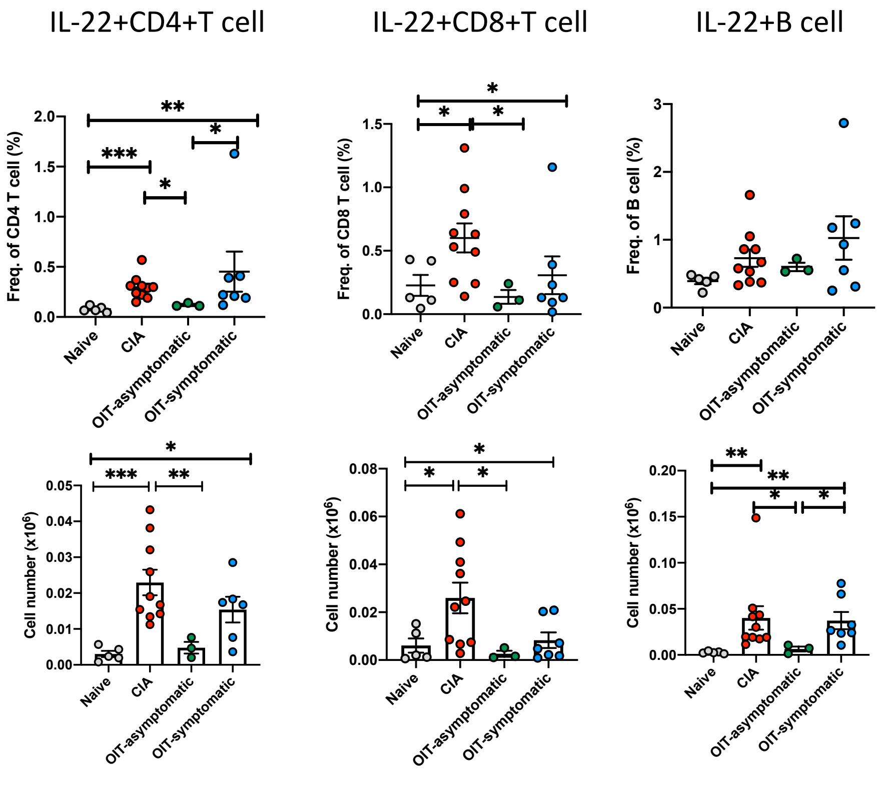

Supplementary figure 4. Raw data for Figure 3F shown as column graphs in naïve, CIA, OIT asymptomatic and OIT symptomatic mice. Cell frequency and cell numbers (evaluated by Flow cytometry) of IL-17+ (a) and IL-22+ (b) CD4 T cells, CD8 T cells and B cells isolated from draining lymph nodes. Each dot represents individual mice, bars show mean value for each group ± SEM, data from 2 independent experiments. Statistical significance was determined by one-way ANOVA; *p < 0.05, ***p < 0.01.

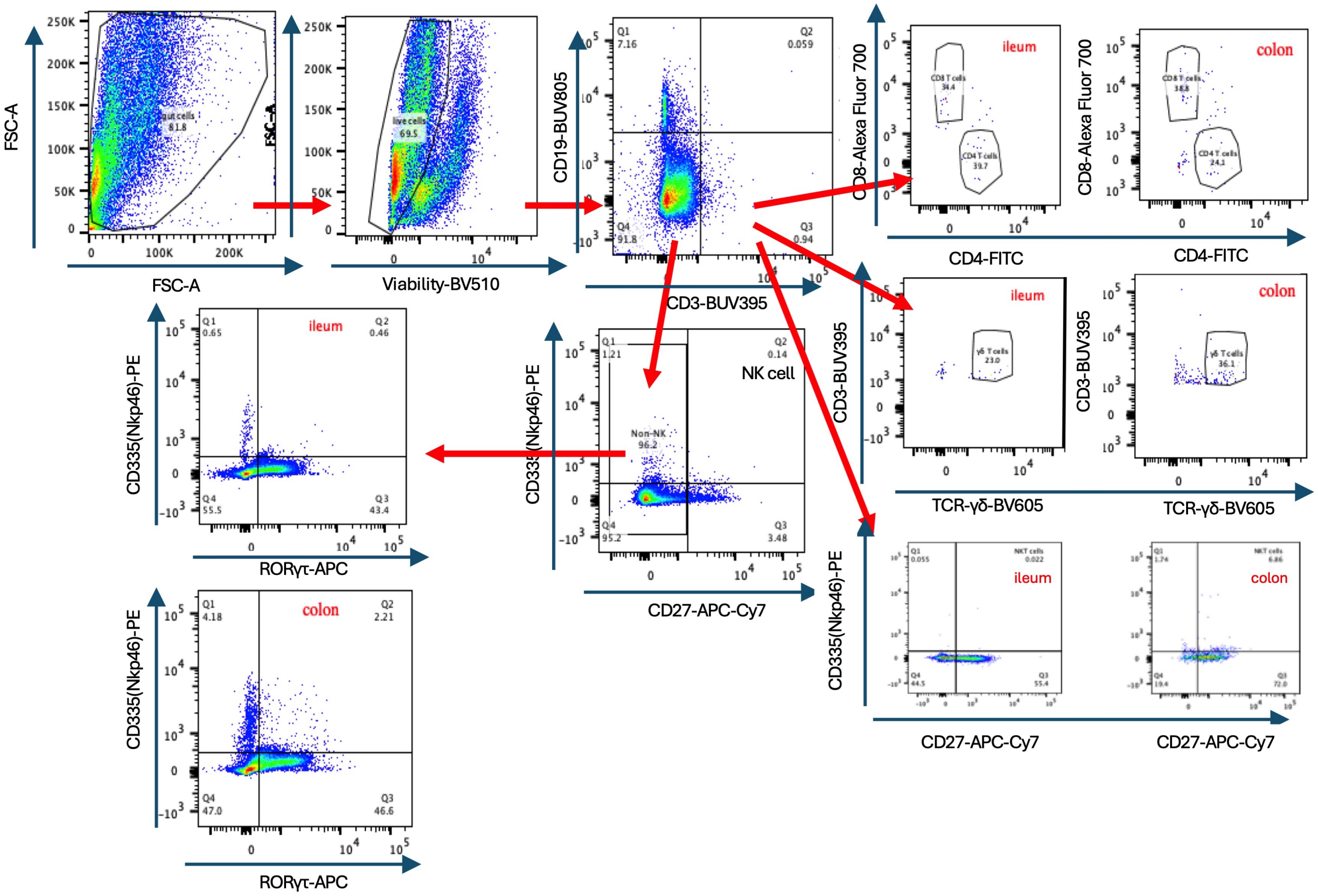

Supplementary Figure 5. Gating strategy for immune system cells in the gut tissue. Gut tissue was digested by collagenase to obtain single cell suspension prior to antibody staining and flow cytometric analysis. Relevant cell populations were first gated on the basis of FSC/SSC analysis and singlet and live-dead cell discrimination using Viability dye (BV510). T cells and B cells were identified by anti CD3 (BUV395) and anti CD19 (BUV805) antibodies respectively. T cells (CD3+) were further gated to identify: CD8 T cells (CD8, Alexa Fluor 700), CD4 T cells (CD4, FITC), γδ Tcell (TCR- γδ, BV605), and NKT cells (CD335+ (PE) CD27+ (APC-Cy7)). Other cells (CD3-CD19-) were gated to further select NK cells by marker CD335 (PE) and CD27 (APC-Cy7). Within CD27- cells ILC3 were identified by expression of CD335 (PE) and RORγτ (APC). IL-17 was stained in BV421 fluorophore, and IL-22 was stained in PerCP-eFlour 710 fluorophore.

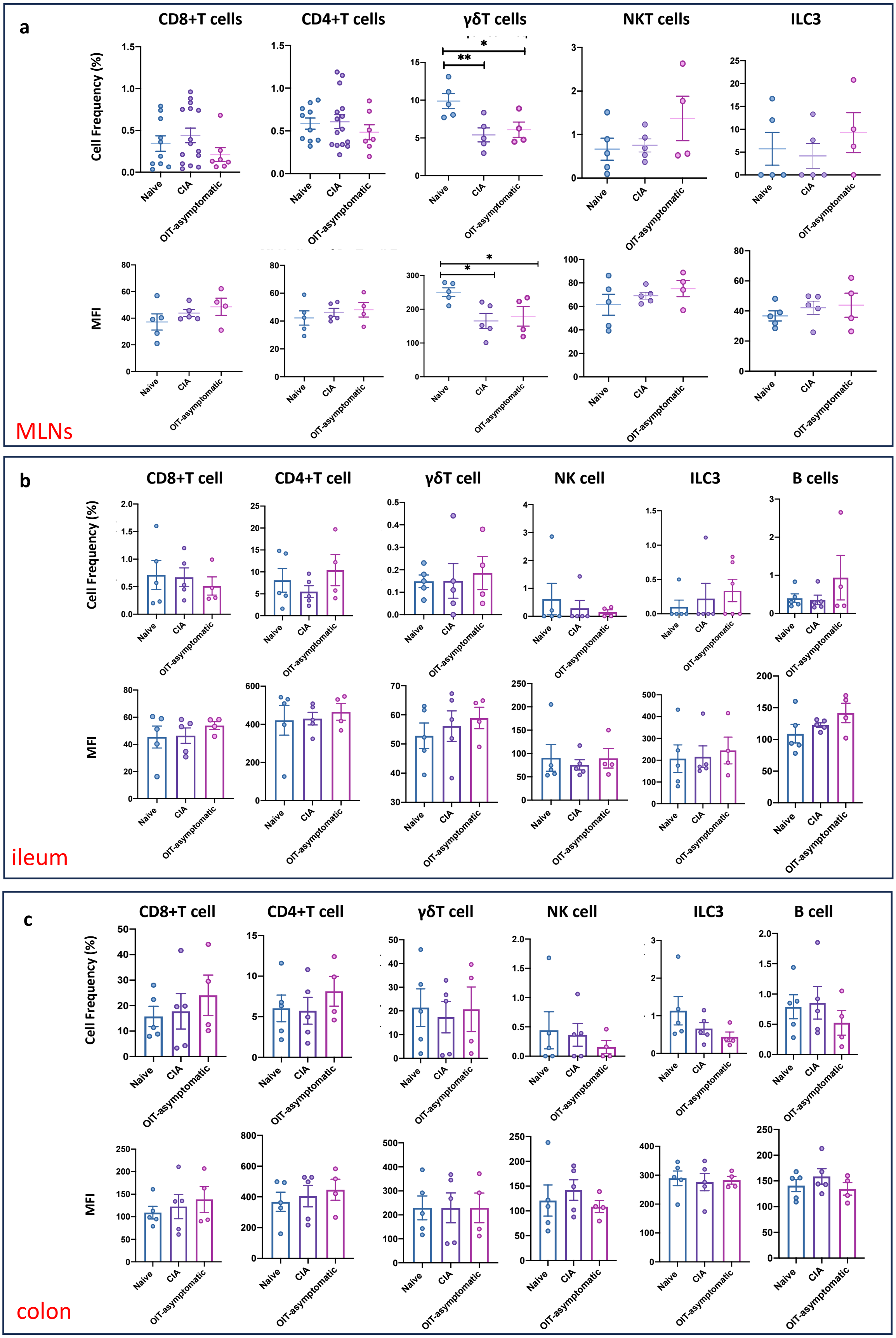

Supplementary Figure 6. Raw data for Figure 5B, 5D and 5F, shown as column graphs in naïve, CIA and OIT asymptomatic mice. Relative frequency for IL-17+ cells and IL-17 mean fluorescence intensity for the indicated cell populations isolated from a) the mesenteric lymph nodes (MLNs), b) ileum tissue and c) colon tissue. Each dot represents individual mice, bars show mean value for each group ± SEM. Statistical significance was determined by one-way ANOVA; *$p < 0.05$.

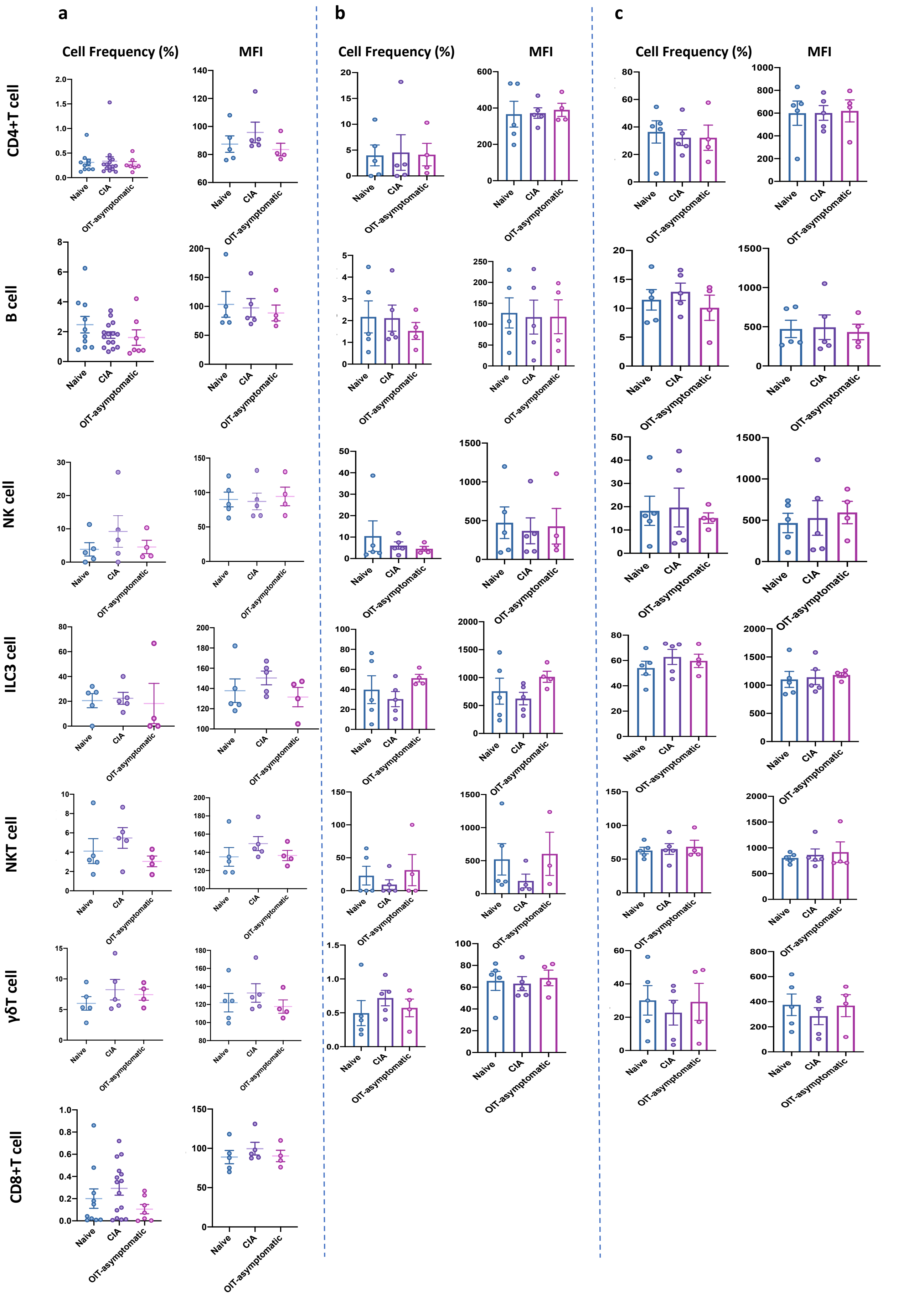

Supplementary Figure 7. Raw data for radar charts in Figure 6B, 6D and 6F, shown as column graphs in naïve, CIA and OIT asymptomatic mice. Relative frequency for IL-22+ cells and IL-22 mean fluorescence intensity for the indicated cell populations isolated from a) the mesenteric lymph nodes (MLNs), b) ileum and c) colon tissue. Each dot represents individual mice, bars show mean value for each group ± SEM.

a

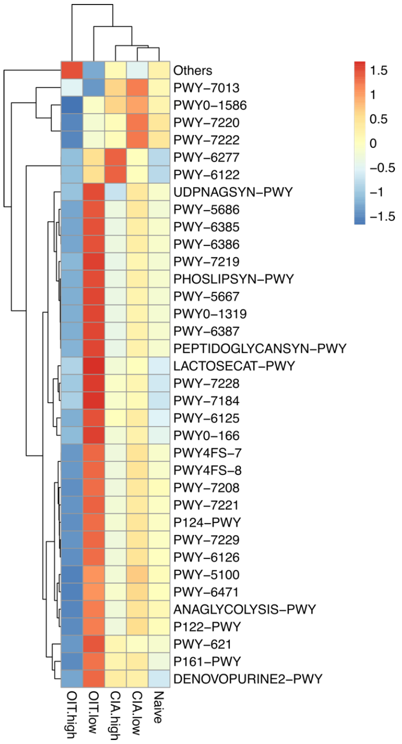

PWY-7013	(S)-propane-1,2-diol degradation
PWY0-1586	peptidoglycan maturation (meso-diaminopimelate containing)
PWY-7220	adenosine ribonucleotides de novo biosynthesis
PWY-7222	guanosine deoxyribonucleotides de novo biosynthesis ii
PWY-6277	superpathway of 5-aminoimidazole ribonucleotide biosynthesis
PWY-6122	5-aminoimidazole ribonucleotide biosynthesis II
UDPNAGSYN-PWY	UDP-N-acetyl-D-glucosamine biosynthesis I
PWY-5686	UMP biosynthesis I
PWY-6385	peptidoglycan biosynthesis III (mycobacteria)
PWY-6386	UDP-N-acetylmuramoyl-pentapeptide biosynthesis II (lysine-containing)
PWY-7219	adenosine ribonucleotides de novo biosynthesis
PHOSLIPSYN-PWY	superpathway of phospholipid biosynthesis III (E. coli)
PWY-5667	CDP-diacylglycerol biosynthesis I
PWY0-1319	CDP-diacylglycerol biosynthesis II
PWY-6387	UDP-N-acetylmuramoyl-pentapeptide biosynthesis I (meso-diaminopimelate containing)
PEPTIDOGLYCANSYN-PWY	peptidoglycan biosynthesis I (meso-diaminopimelate containing)
LACTOSECAT-PWY	lactose degradation I
PWY-7228	superpathway of guanosine nucleotides de novo biosynthesis I
PWY-7184	pyrimidine deoxyribonucleotides de novo biosynthesis I
PWY-6125	superpathway of guanosine nucleotides de novo biosynthesis II
PWY0-166	superpathway of pyrimidine deoxyribonucleotides de novo biosynthesis (E. coli)
PWY4FS-7	phosphatidylglycerol biosynthesis I
PWY4FS-8	phosphatidylglycerol biosynthesis II
PWY-7208	superpathway of pyrimidine nucleobases salvage
PWY-7221	guanosine ribonucleotides de novo biosynthesis
P124-PWY	Bifidobacterium shunt
PWY-7229	superpathway of adenosine nucleotides de novo biosynthesis I
PWY-6126	superpathway of adenosine nucleotides de novo biosynthesis II
PWY-5100	pyruvate fermentation to acetate and lactate II
PWY-6471	peptidoglycan biosynthesis IV (Enterococcus faecium)
ANAGLYCOLYSIS-PWY	glycolysis III (from glucose)
P122-PWY	heterolactic fermentation
PWY-621	sucrose degradation III (sucrose invertase)
P161-PWY	acetylene degradation (anaerobic)
DENOVOPURINE2-PWY	superpathway of purine nucleotides de novo biosynthesis II

b

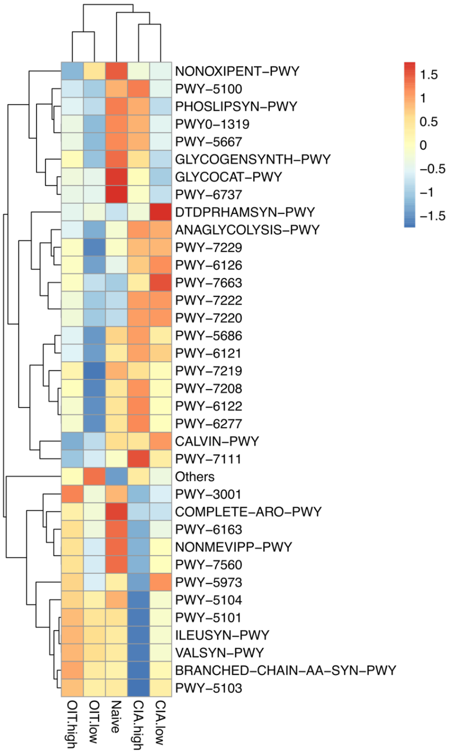

NONOXIPENT-PWY	pentose phosphate pathway (non-oxidative branch) I
PWY-5100	pyruvate fermentation to acetate and lactate II
PHOSLIPSYN-PWY	superpathway of phospholipid biosynthesis III (E. coli)
PWY0-1319	CDP-diacylglycerol biosynthesis II
PWY-5667	CDP-diacylglycerol biosynthesis I
GLYCOGENSYNTH-PWY	glycogen biosynthesis I (from ADP-D-Glucose)
GLYCOCAT-PWY	glycogen degradation I
PWY-6763	salicortin biosynthesis
DTDPRHAMSYN-PWY	dTDP-β-L-rhamnose biosynthesis
ANAGLYCOLYSIS-PWY	glycolysis III (from glucose)
PWY-7229	superpathway of adenosine nucleotides de novo biosynthesis I
PWY-6126	superpathway of adenosine nucleotides de novo biosynthesis II
PWY-7663	gondoate biosynthesis (anaerobic)
PWY-7222	guanosine deoxyribonucleotides de novo biosynthesis II
PWY-7220	adenosine deoxyribonucleotides de novo biosynthesis II
PWY-5686	UMP biosynthesis I
PWY-6121	5-aminoimidazole ribonucleotide biosynthesis I
PWY-7219	adenosine ribonucleotides de novo biosynthesis
PWY-7208	superpathway of pyrimidine nucleobases salvage
PWY-6122	5-aminoimidazole ribonucleotide biosynthesis II
PWY-6277	superpathway of 5-aminoimidazole ribonucleotide biosynthesis
CALVIN-PWY	Calvin-Benson-Bassham cycle
PWY-7111	pyruvate fermentation to isobutanol (engineered)

PWY-3001	superpathway of L-isoleucine biosynthesis I
COMPLETE-ARO-PWY	superpathway of aromatic amino acid biosynthesis
PWY-6163	chorismate biosynthesis from 3-dehydroquinate
NONMEVIPP-PWY	methylerythritol phosphate pathway I
PWY-7560	methylerythritol phosphate pathway II
PWY-5973	cis-vaccenate biosynthesis
PWY-5104	L-isoleucine biosynthesis IV
PWY-5101	L-isoleucine biosynthesis II
ILEUSYN-PWY	L-isoleucine biosynthesis I (from threonine)
VALSYN-PWY	L-valine biosynthesis
BRANCHED-CHAIN-AA-SYN-PWY	superpathway of branched chain amino acid biosynthesis
PWY-5103	L-isoleucine biosynthesis III

Supplementary Figure 8. Prediction of metagenome functions. The bioinformatic software package PICRUSt2 was used to conduce metagenomic function prediction based on 16S rRNA gene in ileum (a) and colon (b) samples. Function prediction was based on KEGG databases.
