# Supplementary material for: Undenatured type II collagen protects against collagen-induced arthritis by restoring gut-joint homeostasis and immunity
Source: Commun Biol. 2024 Jul 3;7:804. doi: 10.1038/s42003-024-06476-z (PMC11222443; doi:10.1038/s42003-024-06476-z)
Supplement: Supplementary file 5 — Reporting summary [file 42003_2024_6476_MOESM5_ESM.pdf]

nature portfolio

Corresponding author(s): Miguel Pineda

Last updated by author(s): Jun 3, 2024

Reporting Summary

Nature Portfolio wishes to improve the reproducibility of the work that we publish. This form provides structure for consistency and transparency in reporting. For further information on Nature Portfolio policies, see our Editorial Policies and the Editorial Policy Checklist.

Statistics

For all statistical analyses, confirm that the following items are present in the figure legend, table legend, main text, or Methods section.

n/a	Confirmed	
☐	☒	The exact sample size (n) for each experimental group/condition, given as a discrete number and unit of measurement
☐	☒	A statement on whether measurements were taken from distinct samples or whether the same sample was measured repeatedly
☐	☒	The statistical test(s) used AND whether they are one- or two-sided<br>*Only common tests should be described solely by name; describe more complex techniques in the Methods section.*
☒	☐	A description of all covariates tested
☐	☒	A description of any assumptions or corrections, such as tests of normality and adjustment for multiple comparisons
☐	☒	A full description of the statistical parameters including central tendency (e.g. means) or other basic estimates (e.g. regression coefficient) AND variation (e.g. standard deviation) or associated estimates of uncertainty (e.g. confidence intervals)
☒	☐	For null hypothesis testing, the test statistic (e.g. F, t, r) with confidence intervals, effect sizes, degrees of freedom and P value noted<br>*Give P values as exact values whenever suitable.*
☒	☐	For Bayesian analysis, information on the choice of priors and Markov chain Monte Carlo settings
☒	☐	For hierarchical and complex designs, identification of the appropriate level for tests and full reporting of outcomes
☒	☐	Estimates of effect sizes (e.g. Cohen's d, Pearson's r), indicating how they were calculated

Our web collection on statistics for biologists contains articles on many of the points above.

Software and code

Policy information about availability of computer code

Data collection	BD FACSDiva™ Software was used for collection of Flow Cytometry data. Zeiss ZEN 2.3 microscopy software was used for image acquisition. Microbial diversity sequencing and analysis was conducted on genomic DNA from the ileum and colon faecal matter. All samples were subjected to paired-end sequencing of 16S target hypervariable regions (amplicons) by Novogene (UK) Company Limited.
Data analysis	GraphPath prism 8 for OS X, Excel version 15.33 and R free software were used for analysis . FACs data were analysed with FlowJo 8.7.3. Quantified libraries for microbiome diversity analysis were pooled and sequenced on PacBio Sequel II/IIe systems and analysis was done using DADA2 and Qiime2 software.

For manuscripts utilizing custom algorithms or software that are central to the research but not yet described in published literature, software must be made available to editors and reviewers. We strongly encourage code deposition in a community repository (e.g. GitHub). See the Nature Portfolio guidelines for submitting code & software for further information.

Data

Policy information about availability of data

All manuscripts must include a data availability statement. This statement should provide the following information, where applicable:

- Accession codes, unique identifiers, or web links for publicly available datasets
- A description of any restrictions on data availability
- For clinical datasets or third party data, please ensure that the statement adheres to our policy

All data supporting the findings of this study are available within the paper and its Supplementary Information. Metagenomics data are deposited at the Sequence Read Archive (SRA) data (Bioproject accession number: PRJNA1117859) , available through NCBI servers available at the following URL https://www.ncbi.nlm.nih.gov/bioproject/PRJNA1117859

Research involving human participants, their data, or biological material

Policy information about studies with human participants or human data. See also policy information about sex, gender (identity/presentation), and sexual orientation and race, ethnicity and racism.

Reporting on sex and gender	n/a
Reporting on race, ethnicity, or other socially relevant groupings	n/a
Population characteristics	n/a
Recruitment	n/a
Ethics oversight	n/a

Note that full information on the approval of the study protocol must also be provided in the manuscript.

Field-specific reporting

Please select the one below that is the best fit for your research. If you are not sure, read the appropriate sections before making your selection.

☒ Life sciences ☐ Behavioural & social sciences ☐ Ecological, evolutionary & environmental sciences

For a reference copy of the document with all sections, see nature.com/documents/nr-reporting-summary-flat.pdf

Life sciences study design

All studies must disclose on these points even when the disclosure is negative.

Sample size	For all experiments, dots in graphs represent one individual mouse, and graphs show the mean value and standard error. Statistical significance was calculated as indicated in the figure legends. A pilot study showed UC-II could induce a 50% protection in CIA mice. The data were used to calculate the number of animals to conduct experiments. Data in figures 1-4 used n=15 (CIA), n=16 (OIT) and n=9 (naïve) from two independent experiments. Figures 5-7 show at least 4 mice per group from one experiment. All data were generated analysing animals individually, from two independent experiments. Microbiome data show individual mice from 5 different groups: Naive (n=4), CIA high scores (n=3), CIA low scores (n=3), symptomatic OIT mice (n=3) and asymptomatic OIT mice (n=3).
Data exclusions	No data were excluded from analysis
Replication	All information regarding biological and experimental replicates are indicated in the figure legends. For ex vivo experiments, cells were isolated from individual mice and cytokine secretion was analysed in technical triplicate for each sample. qPCR experiments were conducted in technical triplicates. For in vivo experiments, all animals were analysed independently, combining data from independent experiments as indicated in figure legends.
Randomization	Before starting experimental models, mice were assigned to individual groups using automatic randomised block design (Excel) under a single blind protocol. Groups were not mixed within cages to prevent microbiota transfer.
Blinding	Clinical and histological scoring were conducted blinded by two independent researchers.

Reporting for specific materials, systems and methods

We require information from authors about some types of materials, experimental systems and methods used in many studies. Here, indicate whether each material, system or method listed is relevant to your study. If you are not sure if a list item applies to your research, read the appropriate section before selecting a response.

Materials & experimental systems

n/a	Involved in the study
	☒ Antibodies
☒	☐ Eukaryotic cell lines
☒	☐ Palaeontology and archaeology
	☒ Animals and other organisms
☒	☐ Clinical data
☒	☐ Dual use research of concern
☒	☐ Plants

Methods

n/a	Involved in the study
☒	☐ ChIP-seq
	☒ Flow cytometry
☒	☐ MRI-based neuroimaging

Antibodies

Antibodies used	1. For Treg panel [need to check company]<br>Viability (eBioscience fixable viability Dye eFluor 780, invitrogen,Cat:65-0865-14), CD3 (APC, BD Biosciences, Clone:17A2, Cat:565643), CD4 (Brilliant Violet 510, Biolegend, Clone:GK1.5, Cat:100449), CD8 (Brilliant Violet605, Biolegend, Clone:53-6.7, Cat:100744), Foxp3 (Brilliant Violet 421, Biolegend, Clone:MF-14, Cat:126419), CD25 (PE,BD Biosciences, Clone: 7D4, Cat:558642), CD39 (PE-CY7,Biolegend,Clone:Duha59, Cat:143806), CD73 (FITC, Biolegend, Clone:TY/11.8, Cat:127220)<br><br>2. Cytokines (Figure 3)<br>Viability (eBioscience fixable viability Dye eFluor 780, invitrogen, Cat:65-0865-14), CD3 (FITC, Biolegend, Clone:145-2C11, Cat:100305), CD19 (Brilliant Violet 421,Biolegend, Clone:6D5, Cat:115537), CD4(AF700, Biolegend,Clone:RM4-4, Cat:116022), CD8 (PE-Cy5, Biolegend, Clone:53-6.7, Cat:100710), IL-17 (PerCP-Cy5.5, eBioscience, Clone:eBio17B7, Cat:45-7177-80), IL-22 (PE, eBioscience, Clone:1H8PWSR, Cat:12-7221-80)<br><br>3. Cytokines (Figure 5 and 6)<br>Viability dye (eBioscience fixable viability Dye eFluor 506, invitrogen, Cat: 65-0866-14), CD19 (BUV805 Rat Anti-Mouse CD19, BD Biosciences, Clone:1 D3, Cat: 749027), CD3 (BUV395 Hamster Anti-Mouse CD3e, BD Biosciences, Clone: 145-2C11, Cat: 563565), CD8 (APC-R700 Rat Anti-Mouse CD8a, BD Biosciences, Clone: 53-6.7, Cat: 564983), TCR γ/δ (Brilliant Violet 605™ anti-mouse TCR γ/δ Antibody, Biolegend, Clone:GL3, Cat: 118129), CD4 (alexa Fluor488, Biolegend, Clone:GK1.5, Cat:100423), RORγt (Alexa Fluor® 647 Mouse Anti-Mouse RORγt, BD Biosciences, Clone: Q31-378, Cat: 562682), CD335(NKp46) (PE anti-mouse CD335 Antibody, Biolegend, Clone:29A1.4, Cat: 137603), CD27 (APC/Fire™ 750 anti-mouse/rat/human CD27 Antibody, Biolegend, Clone:LG.3A10, Cat: 124237), IL-17 (Brilliant Violet 421, Biolegend, Clone:TC-11-18H10.1, Cat:506925), IL-22 (PerCP-eFlour710, eBioscience, Clone:1H8PWSR, Cat:46-7221-82)
Validation	Only well-validated commercial antibodies were used according to the manufacturer's instructions.

Animals and other research organisms

Policy information about studies involving animals; ARRIVE guidelines recommended for reporting animal research, and Sex and Gender in Research

Laboratory animals	Male DBA/1 mice were purchased at 7-8 weeks of age (Envigo; Bicester, UK) and housed and maintained in the Central Research Facility of the University of Glasgow
Wild animals	n/a
Reporting on sex	Only male animals were used in the study.
Field-collected samples	n/a
Ethics oversight	All experiments were approved by, and conducted in accordance with, the Animal Welfare and Ethical Review Board of the University of Glasgow, UK Home Office Regulations and Licenses P8C60C865, I675F0C46 and ID5D5F18C.

Note that full information on the approval of the study protocol must also be provided in the manuscript.

Flow Cytometry

Plots

Confirm that:

☒ The axis labels state the marker and fluorochrome used (e.g. CD4-FITC).

☒ The axis scales are clearly visible. Include numbers along axes only for bottom left plot of group (a 'group' is an analysis of identical markers).

☒ All plots are contour plots with outliers or pseudocolor plots.

☒ A numerical value for number of cells or percentage (with statistics) is provided.

Methodology

Sample preparation	1. For lymph nodes: lymph nodes were smashed into single cell suspensions, washed with PBS twice and stained with dye viability for 30 min. The cells were washed with PBS twice, Fc receptors were blocked for 15 min. After washing with FACs buffer, the cells were stained with specific antibodies as indicated.<br>2. For gut tissue Mouse gut tissue was collected after removal of excess fat and Peyer's patches. Gut was opened longitudinally, washed with PBS and cut into small pieces (around 1 cm). Samples were temporally stored in collection buffer (HBSS 10% FBS). Samples were rinsed with warm HBSS and transfered into a new tube containing wash buffer (HBSS 2mM EDTA) and put in the shaker (37 degrees, 220 rpm) for 15 mins. This step was repeated ftwice. Ileum samples were then transfered to a tube containing 0.5mg/ml of Collagenase IV for tissue digestion. Colon samples were transfered to 0.5 mg/ml of Collagenase IV, 24 µg/ml DNase I. All samples were then incubated in a shaker (37degree, 220 rpm) for 15 min (ileum) or 20 min (colon). After digestion, ice-cold RPMI was added to a final volumen of 30 ml and placed on ice. Samples were filtered samples through 100 µm filter then 70 µm filter, centrifuged for 10mins at 400 g. Supernatant was removed and resuspended in 30 mls ice-cold R10, cemtrigued for a further 10mins at 400 g, when cells were resuspended prior to antibody staining.
Instrument	Data were acquired using a FACS BD LSRII flow cytometer.
Software	FlowJo, LLC analysis software (Tree Star/ BD)
Cell population abundance	No cell sorting was performed.
Gating strategy	1. For Tregs: Relevant cell populations were first gated on the basis of FSC/SSC analysis and singlet and live-dead cell discrimination using Viability dye. Cell populations were gated using isotype controls. T cells were distinguished by expression of CD3, Tregs were identified by their expression of CD8, CD25, and Foxp3. Two subtypes of Tregs were identified by expression of CD39 and CD73.<br><br>2. For cytokines (figure 3): Relevant cell populations were first gated on the basis of FSC/SSC analysis and singlet and live-dead cell discrimination using Viability dye. T cells and B cells were identified by their expression of CD3 and CD19, respectively. Two types of T cells were identified by their marker CD4 and CD8.<br><br>3. For cytokines (figure 5 and 6): Relevant cell populations were first gated on the basis of FSC/SSC analysis and singlet and live-dead cell discrimination using Viability dye. T cells and B cells were identified by their expression of CD3 and CD19, respectively. CD19-CD3- cells were regarded as other cells. T cells could be further identified by markers: CD8 (CD8 T cell), CD4 (CD4 T cell), TCR-γδ (γδ T cell), and Nkp46+CD27+(NKT cell). Other cells could be further identified as follows: Nkp46 +CD27+(NK cell). Nkp46+CD27- cells can be further gated by expression of RORyt, which is ILC3.

☒ Tick this box to confirm that a figure exemplifying the gating strategy is provided in the Supplementary Information.
